# Characterization of a novel microfluidic platform for the isolation of rare single cells to enable CTC analysis from head and neck squamous cell carcinoma patients

**DOI:** 10.1002/elsc.202100133

**Published:** 2022-04-06

**Authors:** Janis Stiefel, Christian Freese, Ashwin Sriram, Sabine Alebrand, Nalini Srinivas, Christoph Sproll, Madita Wandrey, Désirée Gül, Jan Hagemann, Jürgen C. Becker, Michael Baßler

**Affiliations:** ^1^ Fraunhofer Institute for Microengineering and Microsystems IMM Mainz Germany; ^2^ Translational Skin Cancer Research DKTK Partner Site Essen/Düsseldorf West German Cancer Center Dermatology University Duisburg‐Essen, Essen, Germany; German Cancer Research Center (DKFZ) Heidelberg Germany; ^3^ Clinic for Oral and Maxilofacial Surgery Düsseldorf University Hospital Heinrich‐Heine‐University Düsseldorf Germany; ^4^ Department of Otorhinolaryngology/ENT University Medical Center Mainz Germany

**Keywords:** automation, liquid biopsy, microfluidics, precision medicine, tumor diagnostics

## Abstract

Detailed examination of tumor components is leading‐edge to establish personalized cancer therapy. Accompanying research on cell‐free DNA, the cell count of circulating tumor cells (CTCs) in patient blood is seen as a crucial prognostic factor. The potential of CTC analysis is further not limited to the determination of the overall survival rate but sheds light on understanding inter‐ and intratumoral heterogeneity. In this regard, commercial CTC isolation devices combining an efficient enrichment of rare cells with a droplet deposition of single cells for downstream analysis are highly appreciated. The Liquid biopsy platform *CTCelect* was developed to realize a fully‐automated enrichment and single cell dispensing of CTCs from whole blood without pre‐processing. We characterized each process step with two different carcinoma cell lines demonstrating up to 87 % enrichment (n = 10) with EpCAM coupled immunomagnetic beads, 73 % optical detection and dispensing efficiency (n = 5). 40 to 56.7 % of cells were recovered after complete isolation from 7.5 ml untreated whole blood (n = 6). In this study, *CTCelect* enabled automated dispensing of single circulating tumor cells from HNSCC patient samples, qPCR‐based confirmation of tumor‐related biomarkers and immunostaining. Finally, the platform was compared to commercial CTC isolation technologies to highlight advantages and limitations of *CTCelect*. This system offers new possibilities for single cell screening in cancer diagnostics, individual therapy approaches and real‐time monitoring.

**Abbreviations**
μCS: microfluidic fluorescence‐activated cell sorting
Ab: antibody
BSA: bovine serum albumin
CFSE: carboxyfluorescein succinimidyl ester
C_T_
: threshold cycle
CTC: circulating tumor cell
ctDNA: circulating tumor DNA
DTC: disseminated tumor cell
EDTA: ethylenediaminetetraacetic acid
EMT: epithelial‐mesenchymal transition
EpCAM: epithelial cell adhesion molecule
FDA: Food and Drug Administration
HNSCC: head and neck squamous cell carcinoma
IMS: immunomagnetic separation
MCF‐7: Michigan Cancer Foundation 7
PBMC: peripheral blood mononuclear cells
PE: phycoerythrin
pEMT: partial/post‐EMT
pTNM staging: histopathologic tumor‐nodes‐metastasis staging
RT‐qPCR: real time quantitative polymerase chain reaction
SCL‐1: Squamous cancer line 1
WBC: white blood cell

## 1 INTRODUCTION

In the field of liquid biopsy, research on circulating tumor DNA (ctDNA) combined with the detection and analysis of circulating tumor cells (CTCs) has developed into an auspicious minimal invasive tool for the early detection in personalized medicine for tumor patients [1]. Due to the fact that CTCs play an important role in cancer metastasis as the main cause of tumor death, the number of CTCs in a blood sample is considered an independent prognostic factor for the overall survival [2]. In addition to the CTC count particularly on the hunt for the primary tumor in early stage cancer, CTC analysis refines the selection, adaptation and even development of therapies. Although the overall principle of complex metastasis, from the intravasation of CTCs into the circulatory system, arrest and extravasation through vascular walls into distant tissues to the final proliferation of cells to micrometastases, is understood, future intensive study on CTCs and their subtypes will result in much deeper knowledge of these multifaceted processes. Recent studies show that the characterization of CTCs and their subpopulations, that is, disseminated tumor cells (DTC), metastasis‐initiating cells and even dual‐positive cells (CD45^+^/EpCAM^+^) helps to understand the metastatic behavior of tumors and to deduce prognostic predictions and diagnostic statements [3, 4].

When developing isolation technologies for these cells, their rarity in a blood sample is the biggest hurdle to overcome. Besides billions of healthy blood cells, only one to hundreds tumor‐associated cells can be found per ml blood [1]. Systems that are able to enrich CTCs from blood well‐balanced between sensitivity and specificity, are therefore highly appreciated. In addition to the enrichment, such systems should exhibit a high capture efficiency, high isolation purity, and ideally the ability to handle a high sample volume in the shortest time possible [5].

Available techniques are based on two major principles: physical methods such as filtration and density dependent techniques or biochemical immuno‐affinity dependent methods using ligand‐surface interactions with bound antibodies or antibody‐bound magnetic beads for the enrichment of CTCs.

The CellSearch system (Menarini Silicon Biosystems) uses magnetic beads that are biofunctionalized against epitopes of tumor‐associated EpCAM (Epithelial cell adhesion molecule) to enrich CTCs from patient blood [6, 7]. It has been established to correlate the number of isolated EpCAM^+^ cells in a blood sample with the overall survival prediction of breast cancer, metastatic colorectal cancer and prostate cancer patients [8, 9, 10]. Nevertheless, CTC research in other cancer entities like head and neck squamous cell carcinoma (HNSCC) remains comparably stagnant. With an annual incidence of almost a million new cases and 450,000 deaths worldwide, HNSCC ranks however the fifth most common cancer [11]. Besides carcinogenic polymorph dispositions, smoking, alcohol abuse, and an infection with human papillomavirus correlate with head and neck cancers [12]. In two third of HNSCC patients, initial diagnosis of the primary tumor goes hand in hand with the discovery of adjacent metastatic lymph nodes [13]. Increased mortality is associated with the abundant presence of micrometastases, whereas the 5‐year survival rate of patients with distant metastatic sites sinks below 35 % [14]. HNSCC cells that undergo epithelial‐mesenchymal transition (EMT) deregulate epithelial characteristics like cell adhesion and enhance invasive migration. EMT is a dynamic reversible process correlating with the development of stem cell properties and fostering metastasis. This fact stresses the importance of an in‐depth understanding of metastatic metabolisms, including the heterogeneity of disease‐driving CTCs, to pave the way for innovative therapeutics.

**FIGURE 1:**
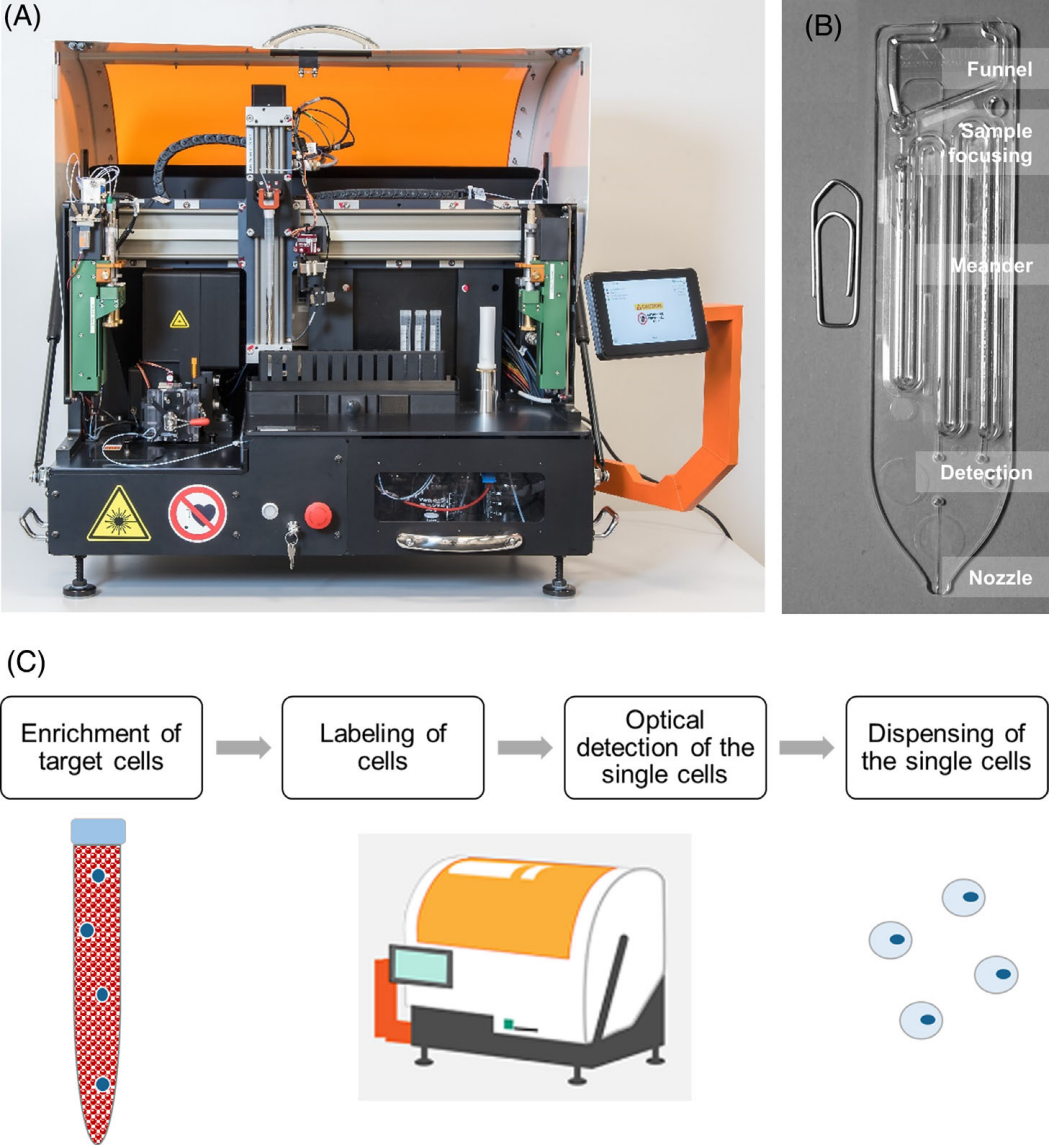
*CTCelect* system, microfluidic chip and process conception. (A) The benchtop device consists of an immunomagnetic enrichment module (right‐sided) and a microfluidic fluorescence‐activated cell sorting (μCS) subunit (left‐sided). The fully‐automated isolation process is user‐controlled via touchscreen. Sample handling and transfer is managed by a pipetting robot. (B) The *CTCelect* chip is placed in the chip holder of the cell sorting subunit and disposed after isolation. The chip consists of a reservoir funnel for the cell suspension, a hydrodynamic focusing channel, a detection zone and a nozzle for cell dispensing. (C) *CTCelect* concept for single cell dispensing from 7.5 mL samples

The aim of the present work was to characterize a fully‐automated system in detail which is customizable for any tumor type with a reliable capture rate and CTC purity facilitating access to molecular diagnostics. The presented *CTCelect* system combines immunomagnetic enrichment from 7.5 ml whole blood, microfluidic fluorescence‐activated cell sorting (μCS) and single cell dispensing through a microfluidic disposable cartridge independently of the tumor entity (Figure 1) in one fully‐automated benchtop device. This study aimed to evaluate the functionality and the diverse applicability of *CTCelect* by means of cutaneous squamous cell carcinoma and mamma carcinoma cell line models and to present data of dispensed single tumor cells from HNSCC patient blood.

**FIGURE 2:**
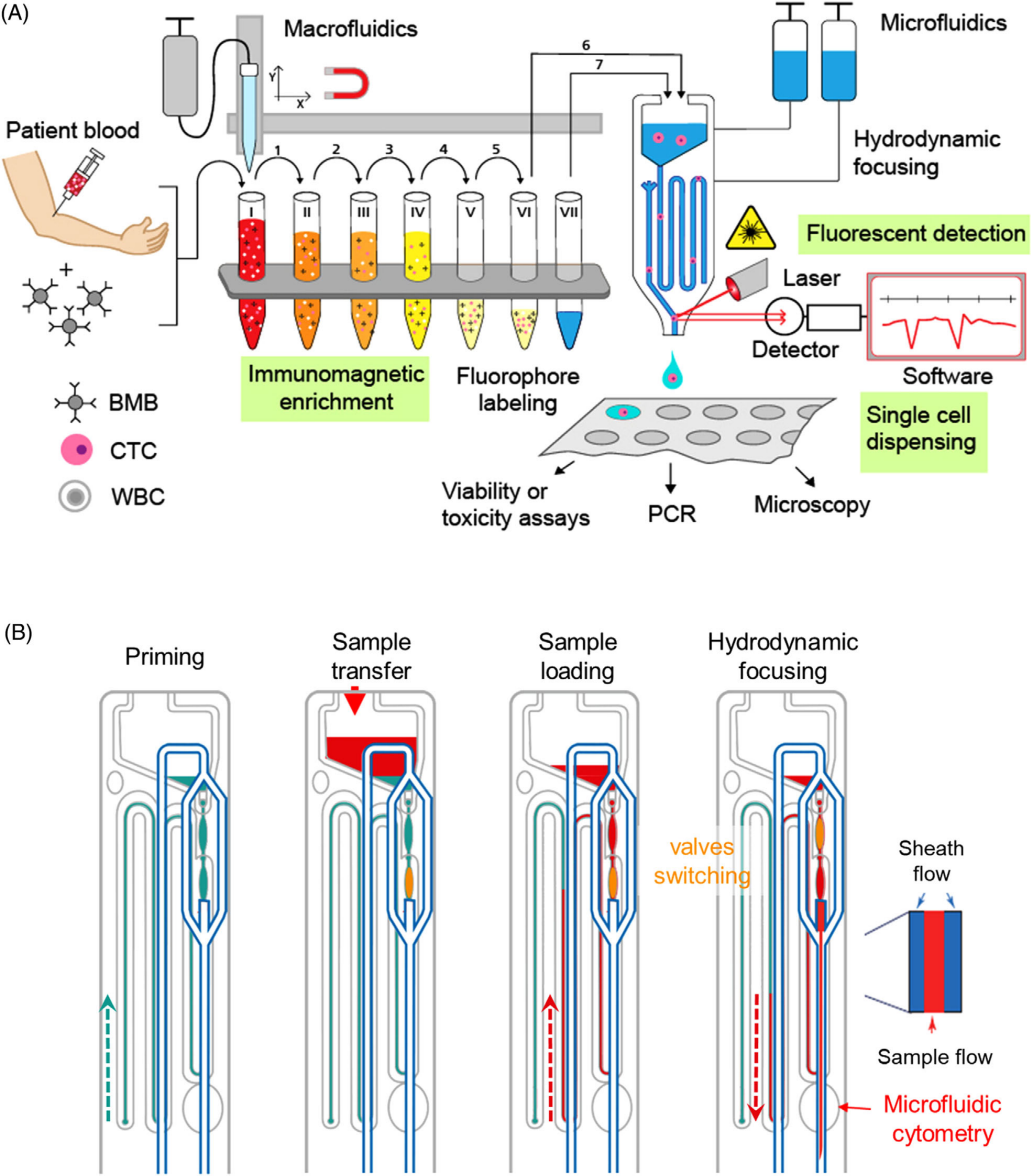
Automated workflow of *CTCelect* single cell isolation. (A) BMB: Biofunctionalized magnetic beads; CTC: Circulating tumor cell; WBC: White blood cell. 7.5 mL Patient blood sample is incubated with BMB to capture CTCs. Positive selection of tumor cells with subsequent depletion of WBCs and fluorophore‐coupled labeling takes place in the enrichment module of the device. The enriched sample is then transferred to the cell sorting subunit into the microfluidic chip for cell sorting and single cell dispensing in microliter droplets. Singularized CTCs can be administered to transcriptomic and cytobiological downstream analyses. (B) Functioning principle of the microfluidic CTCelect chip. The chip meander is primed with sheath flow buffer. Subsequently, the enriched sample is transferred in the chip funnel and loaded in the meander. By switching the valve positions, the sample can then be pushed back in the detection channel and hydrodynamically focused for cytometry using two outer sheath flows

## 2 MATERIALS AND METHODS

If not otherwise indicated, reagents and supplements were purchased at Thermo Fisher Scientific, Darmstadt, DE.

### 2.1 Cell lines

MCF‐7 breast cancer cells were purchased from AdnaGen (Langenhagen, DE) and cultured in RPMI1640 medium with L‐glutamine (Capricorn Scientific, Ebsdorfergrund, DE) supplemented with 10 % fetal calf serum (FCS; Merck, Darmstadt, DE). The SCL‐1 squamous cell carcinoma line was kindly provided by Dr. Petra Boukamp (DKFZ, Heidelberg, DE) [15] and cultured in Gibco DMEM (low glucose, pyruvate) medium supplemented with 10 % FCS. Cell lines were split at subconfluence and incubated at 37°C in a humidified atmosphere in the presence of 5 % CO_2_.

### 2.2 Blood samples

Whole blood bags from healthy donors were obtained from the local Blood Transfusion Center (University Medical Center Mainz, DE) in 500 mL CompoFlex blood bags (Fresenius Kabi, Bad Homburg, DE) with CPD‐1 anticoagulant and stored at room temperature for a maximum of three days. Patient samples were collected at the Tumor Center of Western Germany (University Hospital Essen, DE) from a HNSCC patient with increasing metastasis in adjacent lymph nodes (pTNM staging: pT2pN3b), at the Department of Otorhinolaryngology/ENT, (University Medical Center Mainz, DE; pTNM staging n.a.) and processed within 24 h after blood draw. Informed consent was obtained from the patients and approved by the ethical committee vote at the Medical Faculty of the Heinrich‐Heine‐University Düsseldorf (ref.no. 3090; 2016) and by the local ethics committee in Mainz (ref.no. 837.485.15 (10253); 2016).

**PRACTICAL APPLICATION**

Still today standardized therapeutic guidelines like relatively broadband chemotherapy are followed depending on the primary tumor entity neglecting the metastatic profile of systemic cancer. On the downside, disseminated and circulating tumor cells of many cancers show intratumoral heterogeneity and cause disease relapse years after surgery. Hence, easy‐to‐use platforms, like the show‐cased CTCelect device, to isolate these rare cells in a completely automated way and analyze them on a single cell level will be of high significance as predictive measures for therapeutic success to accompany cancer treatment. The microfluidic chip‐based cell sorting unit was implemented into a one‐step device for user‐friendly handling. Studies with the system could therefore directly provide new insights both in therapy monitoring as clinical application and in basic research of tumor biology to unravel metastatic processes.

### 2.3 Immobilization of biotinylated antibodies on streptavidin coupled magnetic microbeads

The immunomagnetic separation (IMS) of CTCs from whole blood required tumor‐specific coating of immunomagnetic beads with biotinylated monoclonal mouse anti‐human EpCAM (CD326; 20 µg/mL) antibody 1B7. Various beads with different sizes ranging from 1 to 4.5 µm diameter and different surface properties (tosyl‐activated, hydrophobic; carboxylic acid, hydrophilic) were tested with SCL‐1 cells in our preliminary work and the herein used Invitrogen Dynabeads MyOne Streptavidin T1 (10 mg/ml) with a binding capacity of 400 pmol biotinylated peptides per mg beads enabled the best recovery rates in our setting. These microbeads indicate a low sedimentation rate and high binding capacity due to their small diameter and are hence ideal for automated enrichment. EpCAM antibody was immobilized on the bead surface with an extended incubation time of 1 hour. After immobilization, magnetic beads were added a saturated biotin/PBS solution for 30 min with gentle rotation of the tube to block free streptavidin binding sites and prevent clumping. After an additional washing step, anti‐EpCAM magnetic beads (hereinafter abbreviated as EpCAM beads) were stored in PBS/0.1 % BSA at 4°C for several weeks.

### 2.4 Determination of *CTCelect* recovery rates of cultured tumor cells

To evaluate the *CTCelect* enrichment efficacy, recovery rates of cultured tumor cells after automated IMS from medium and whole blood were determined. Robust cancer cell models were required and MCF‐7 and SCL‐1 cells are a suitable candidate for immunomagnetic enrichment to prevalidate the functionality of the platform. We tested both cell lines to have high EpCAM expression (see Supporting Information S1). Cells were stained using the CellTrace CFSE Cell Proliferation Kit (CFSE) according to the manufacturer instructions. 15 mL sized ROTILABO centrifuge tubes without rim (Carl Roth, Karlsruhe, DE) are most suitable for the tube holder of *CTCelect* and were used in all the experiments. Culture medium or whole blood from healthy donors was aliquoted in volumes of 7.55 mL medium or 7.5 mL blood and spiked with different numbers of stained tumor cells, respectively (tube I). 100 µl EpCAM beads (1 mg) were added to medium samples, blood samples contained 150 µl EpCAM beads (1.5 mg), both resulting in a total volume of 7.65 mL and a final beads concentration of 0.13 ‐ 0.2 mg/ml. Subsequently, tubes II, III and IV were prepared with 5 mL buffer 1 (PBS/20 % FCS, 2 mM EDTA) and two tubes (V‐VI) were filled with 0.5 mL buffer 2 (PBS/0.1 % BSA, 2 mM EDTA).

To start the automated enrichment, tubes I‐VI and a 10 mL pipet tip were placed in the holders of the *CTCelect* device as described in Table 1 and the process was initiated on the touchscreen. Before moving on to the next tube, each washing step was alternated with magnetic capture of bead‐bound tumor cells and residual beads in the pipet tip (see Supp. S2). The processed samples were manually separated in a magnet separator for evaluation purposes. Supernatant buffer was discarded and the bead cell pellet was resuspended in 50 µl PBS. CTC counts were determined in a Neubauer counting chamber using fluorescence microscopy.

**TABLE 1:** Automation process of the complete *CTCelect* single cell isolation

**Process**	**Location**	**Volume [mL]**	**Contents**	**Description (Duration)**
IMS	Tube I	7.65	Spiked medium/whole blood; immuno‐magnetic beads	Incubation (30 min) with gentle mixing
Washing	Tube II	5	Buffer 1	Wash off beads‐cell complexes; leukocyte reduction
	Tube III	5		
	Tube IV	5		
Staining	Tube V	0.3	Buffer 2	Ab staining, volume reduction
Transfer	Tube VI	0.3	Transfer buffer	Transfer to *CTCelect* chip
Reservoir	Tube VII	0.5	Transfer buffer	Overlay on residual sample
μCS	*CTCelect* chip	0.25 max	Transfer buffer/PBS	Laser‐based detection; Single cell dispensing

### 2.5 Automated *CTCelect* single cell isolation of cultured tumor cells and CTCs from patient blood

Fully‐automated *CTCelect* single cell isolation was evaluated with cultured tumor cells from medium and whole blood (2.5.a). The automated workflow is depicted in Figure 1C. Further, potential CTCs from HNSCC patient blood were isolated by means of the *CTCelect* single cell dispensing unit after manual pre‐enrichment using detachable beads (2.5.b) and complete isolation (2.5.c). Experiments were performed at different sites in Germany which was easily manageable because both sites have their own CTCelect device to address time‐sensitivity. In total, the team built three devices.

- The functionality of the developed assay was confirmed with spike‐in experiments using intracellular CFSE dye. 20 CFSE pre‐stained tumor cells were added to 7.55 mL medium or 7.5 mL whole blood from healthy donors.
- To confirm epithelial/pEMT origin of HNSCC cells from patient blood, single cell dispensing was investigated after manual pre‐enrichment from peripheral blood using detachable Invitrogen™ CELLection^TM^ Epithelial Enrich anti‐CD326 and ‐CD51, ‐CD61, ‐CD106 immunomagnetic beads (Miltenyi Biotec, Bergisch Gladbach, DE) combined with phycoerythrin‐coupled monoclonal CD326 and CD51 antibody (EpCAM‐PE Ab/Integrin α‐V‐PE Ab; both Biolegend, Koblenz, DE). Pre‐enrichment was performed manually to detach the beads from the cells. The respective research group has access to downstream NGS technologies that is, Nanostring, 10x Genomics that require bead‐free cells after single cell dispensing.
- Complete single cell isolation was verified with 7.5 mL actual patient blood with EpCAM‐PE Ab 1B7 (1:30 in buffer 2). Single cell isolation was performed as described in Table 1 and outlined in Figure 2A.

The enriched and labeled sample was then transferred to the disposable *CTCelect* chip with a final volume of 300 µL in the chip reservoir. Similar to a conventional flow cytometer, the *CTCelect* cell detection is based on the principle of hydrodynamic focusing and optical fluorescence detection (Figure 2B). Microfluidic handling of the chip was managed with a system of two syringes directing sample and sheath flow. The sample flow consisted of a self‐formulated transfer buffer, while sheath flow and dispenser were supplied with PBS. The maximum sample volume (250 µl) was loaded in the chip meander and μCS was initiated by the *CTCelect* software for the first measurement. Fluorescent cells were dispensed in the cavities of a 96‐well plate if matching the given real‐time peak analysis criteria of the fluorescence detection algorithm. Subsequently, 500 µL transfer buffer was pipetted from tube VII to the chip funnel to overlay the residual 50 µL sample and to avoid air bubble formation in the chip channels. The rest of the sample was then loaded in the chip for a second measurement cycle. Dispensed single cell droplets on the 96‐well plate were evaluated using fluorescence microscopy (10x objective) in the FITC and TRITC channel or RT‐qPCR.

### 2.6 Characterization of blood cell contamination in CTC enriched samples

Especially on single cell level, white blood cell (WBC) contamination in tumor cell isolates complicates molecular downstream analyses. The number of CD45+ WBCs after IMS was determined by flow cytometry. Wash buffers were prepared and placed in the tube holder of the *CTCelect* device as described in Table 1 and 150 µL EpCAM beads were added to 7.5 mL whole blood before initiating automated IMS. The final sample was separated in a magnet separator. The supernatant was discarded and the bead cell pellet was stained with CD45‐FITC Ab solution (diluted 1:5 in buffer 2; BD Biosciences, Franklin Lakes, USA). Flow cytometry was performed using the BD Accuri C6 flow cytometer. Gates were set with CD45‐FITC stained beads as negative control and peripheral blood mononuclear cells (PBMCs) from a buffy coat as positive control (see Supp. S3). CD45+ cell counts after IMS were obtained from two samples of different healthy donors and the average WBC contamination with standard deviation (SD) was calculated.

### 2.7 Single cell RT‐qPCR of isolated CTCs

To confirm the CTC/DTC transcriptomic character in the single cell isolates from HNSCC patient blood, two‐step real time quantitative PCR (RT‐qPCR) of target RNAs for EpCAM, Integrin α‐V and Stratifin was performed. β‐actin (RPLP0) served as housekeeping RNA control. RNA was extracted from dispensed droplets immediately after *CTCelect* isolation using a single cell RNA purification kit (GenElute, Merck, DE) and reverse transcribed with the Invitrogen SuperScript double stranded cDNA synthesis kit. qPCR was conducted in triplicates (Invitrogen^TM^ SYBR Green PCR Master Mix) on a single cell cDNA per well in a 384 well cycler (BioRad CFX384 Touch Real‐Time PCR Detection System, Feldkirchen, DE) and individual threshold cycle values (C_T_) were obtained. Relative mRNA expression was calculated using the ΔC_T_ method. Primer sequences are available upon request.

### 2.8 Immunostaining of isolated CTCs

CTCelect single cell isolates were pooled together to assess tumor origin of the isolated cells using immunostaining. The sample was stained with fluorescent CD45‐FITC antibody (1:1000; BD Biosciences, Franklin Lakes, USA) to label leukocytes. Subsequently, the sample was resuspended in EndoPrime medium (Capricorn Scientific, Ebsdorfergrund, DE) and transferred to an ibidi 8‐well slide to set overnight. Cells were centrifuged to the bottom of the slide by a CytoSpin device (300 x g, 10 min) and fixed by using 4% paraformaldehyde for 15 minutes. After further washing steps, cells were permeabilized (0.1% Triton X in PBS, 5 minutes) and stained with nuclear dye Hoechst33342 (1:1000) for 20 min. Alternated with several washing steps, unspecific binding was blocked for 30 min using 0.5% BSA in PBS and the sample was incubated with polyclonal anti‐Zonula occludens‐1 primary antibody 40–2200 (ZO‐1; 1:200, 1 h) and anti‐rabbit Alexa Fluor 633 secondary antibody (1:500, 1h).

### 2.9 Statistical analyses

Each experiment was repeated at least three times. Data is depicted as means with SD and statistical analysis was done using GraphPad PRISM 8.2.0 for Windows (GraphPad Software, San Diego, California USA, www.graphpad.com). P values of two‐tailed unpaired t‐tests were reported as not significant (ns) when P^ns^ > 0.05 and as significant when P* ≤ 0.05.

## 3 RESULTS

### 3.1 *CTCelect* enrichment performance

**FIGURE 3:**
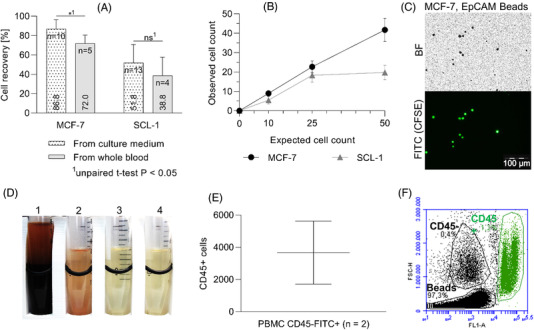
*CTCelect* enrichment performance. (A) Recovery rates of MCF‐7 and SCL‐1 cells after automated IMS from culture medium (black dotted) and whole blood (gray). 10 to 50 cells were spiked into the samples, respectively. Cells were stained with CFSE and enriched with 1 µm EpCAM Beads. Recovery rates of all experiments were averaged and are shown as means with SD and statistical analysis using a two‐tailed unpaired t‐test *P* < 0.05. (B) Observed MCF‐7 (black circles) and SCL‐1 (gray triangles) cell counts after *CTCelect* enrichment from medium were plotted as means with SD against the expected cell count (n ≥ 3). (C) Bright field (BF) and fluorescence microscopy of CFSE stained and EpCAM bead‐bound single MCF‐7 cells (scale bar: 100 µm). (D) Blood residues (1) and wash buffers with blood component waste (2‐4) of subsequent *CTCelect* enrichment process steps in a magnetic separator. In this case almost no loss of sample/beads occurred. Otherwise, beads would be visible as brown accumulations on the backside of the tube. (E) Cell count of CD45^+^ blood cell contamination after *CTCelect* enrichment with EpCAM beads from 7.5 mL donor blood. Contamination was determined via flow cytometry with CD45‐FITC Ab staining. (F) Flow cytometry data of a 7.5 mL blood sample after *CTCelect* enrichment. The scatter plot shows green fluorescence (FL1) on the x‐axis against forward scatter (FSC) particle size on the y‐axis. Populations of beads, CD45^−^ and CD45^+^ cells (green) are distinguished in circles

The functionality of the *CTCelect* enrichment unit was investigated by spike‐in experiments with different tumor cell lines in culture medium. 7.55 mL medium aliquots were spiked with 10, 25, and 50 CFSE‐stained breast cancer cells (MCF‐7) or squamous cell carcinoma cells (SCL‐1). EpCAM beads were added and the samples were processed in the *CTCelect* enrichment unit with the above described protocol (Table 1). After evaluating the device functionality in culture medium, comparable experiments were performed in 7.5 mL whole blood from healthy donors. Following the protocol, all buffers were placed in the device and the enrichment assay was started. Samples were automatically enriched and washed from blood components, subsequently (Figure 3D).

Cell counts after *CTCelect* enrichment were determined visually using fluorescence microscopy. Intact cells were identified as round‐shaped, green fluorescing objects with a bead‐bound surface (Figure 3C). MCF‐7 cells were automatically enriched with an 86.8 ± 9.7 % recovery rate from medium and with 72 ± 8.4 % from whole blood, hence the enrichment efficacy from blood was lower than from culture medium with only minor significance (*P* ^*^  =  0.0122).

Further, 51.8 ± 18.9 % of SCL‐1 cells were recaptured from medium and 38.8 ± 18.9 % from donor blood after automated IMS with EpCAM beads, meaning enrichment efficacy between blood and medium was not significantly different (P^ns^  =  0.2437). The mean recovery rates of all experiments are shown in dependency of the cell line, respectively (Figure 3A). Observed cell counts after *CTCelect* enrichment from medium were plotted against the expected cell count of 10, 25 and 50 spiked cells and summarized in Figure 3B. Recovery rates of MCF‐7 cells showed a nearly linear correlation, independently of the expected cell count between 10 to 50 cells. Up to 25 expected cells, automated recovery of SCL‐1 cells followed a similar stable linearity but resulted in a flattened curve at an expected cell count above 25 cells input. Less than half of 50 spiked SCL‐1 cells could be automatically enriched with EpCAM beads.

As blood cell contamination is a disruptive factor for CTC downstream molecular analyzes, the reduction of CD45^+^ PBMCs in CTC enriched samples was determined additionally. Therefore, processed blood samples were stained with CD45‐FITC Ab after automated *CTCelect* enrichment from 7.5 mL donor whole blood. On average, 3,665 counts of PBMCs were measured in a total volume of 300 µL via flow cytometry (Figure 3E‐F; population indicated in green). Comparing to the CD45^+^ cell count in blood sample #1 (19*10^6^ CD45^+^ cells; data not shown), the number of CD45^+^ cells was significantly reduced using the *CTCelect* enrichment.

### 3.2 Automated single cell isolation from culture medium and donor blood

**FIGURE 4:**
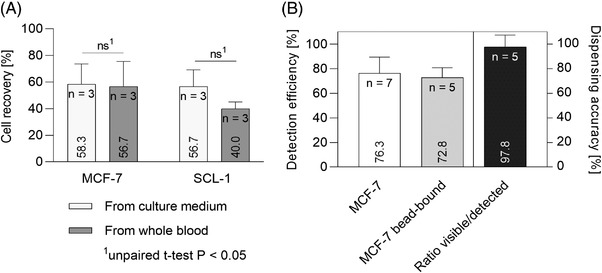
*CTCelect* single cell detection, dispensing and isolation from 7.5 mL samples. (A) Recovery rates of MCF‐7 and SCL‐1 cells after automated single cell isolation from culture medium (light gray) and whole blood (dark gray). 20 CFSE stained cells were spiked into 7.5 mL samples and incubated with 1 µm EpCAM beads. Pre‐enriched cells were automatically singularized and dispensed in droplets after μCS in the *CTCelect* chip. Recovery rates of all experiments were determined with fluorescence microscopy of the droplets and are displayed as means ± SD (ns; two‐tailed unpaired t‐test P < 0.05). (B) Detection efficiency and dispensing accuracy of *CTCelect* μCS. 50 CFSE stained, unbound and bead‐bound MCF‐7 cells were directly spiked in the *CTCelect* chip for single cell isolation. Droplets were microscopically screened for single cells and detection efficiency was averaged by the observed cell count in dependency of the spiked cell number. Dispensing accuracy was calculated dividing the actual number of visible cells in the droplets by the number of events detected by the software

Complete automated single cell isolation was evaluated by means of spike‐in experiments of 20 MCF‐7 and SCL‐1 cells in culture medium and whole blood from healthy donors. *CTCelect* device functionality was confirmed using CFSE staining according to Table 1. Regarding the MCF‐7 cell line model with CFSE dye, 58.3 ± 15.3 % of single cells were counted after isolation from culture medium and 56.7 ± 19 % were detected after isolation from whole blood with comparable recovery efficiency. With the same experimental setup, SCL‐1 cells were automatically isolated at rates of 56.7 ± 18.9 % (medium) and 40.0 ± 5.0 % (whole blood) recovery (Figure 4A). No significant differences in enrichment efficiencies between ‘from medium’ or ‘from donor blood’ is seen for both SCL‐1 (P^ns^  =  0.1807) and MCF‐7 cells (P^ns^  =  0.8870).

More specifically detailed, the detection efficiency and dispensing accuracy of the peak analysis software and microfluidic dispensing through the chip was investigated (Figure 4B), Direct processing of CFSE stained MCF‐7 cells led to visible detection of 76.3 % unbound cells and 72.8 % bead‐bound cells without significant difference. Furthermore, a very high dispensing accuracy of 97.8 % was determined as the ratio between visible bead‐bound cells in the droplets and the detected events in the software, respectively. The microfluidic parameters and flow properties of single cells were intensively studied in our preliminary work to set the dispensing criteria (data not shown).

### 3.3 Verification of epithelial/pEMT origin of HNSCC cells from patient blood

**FIGURE 5:**
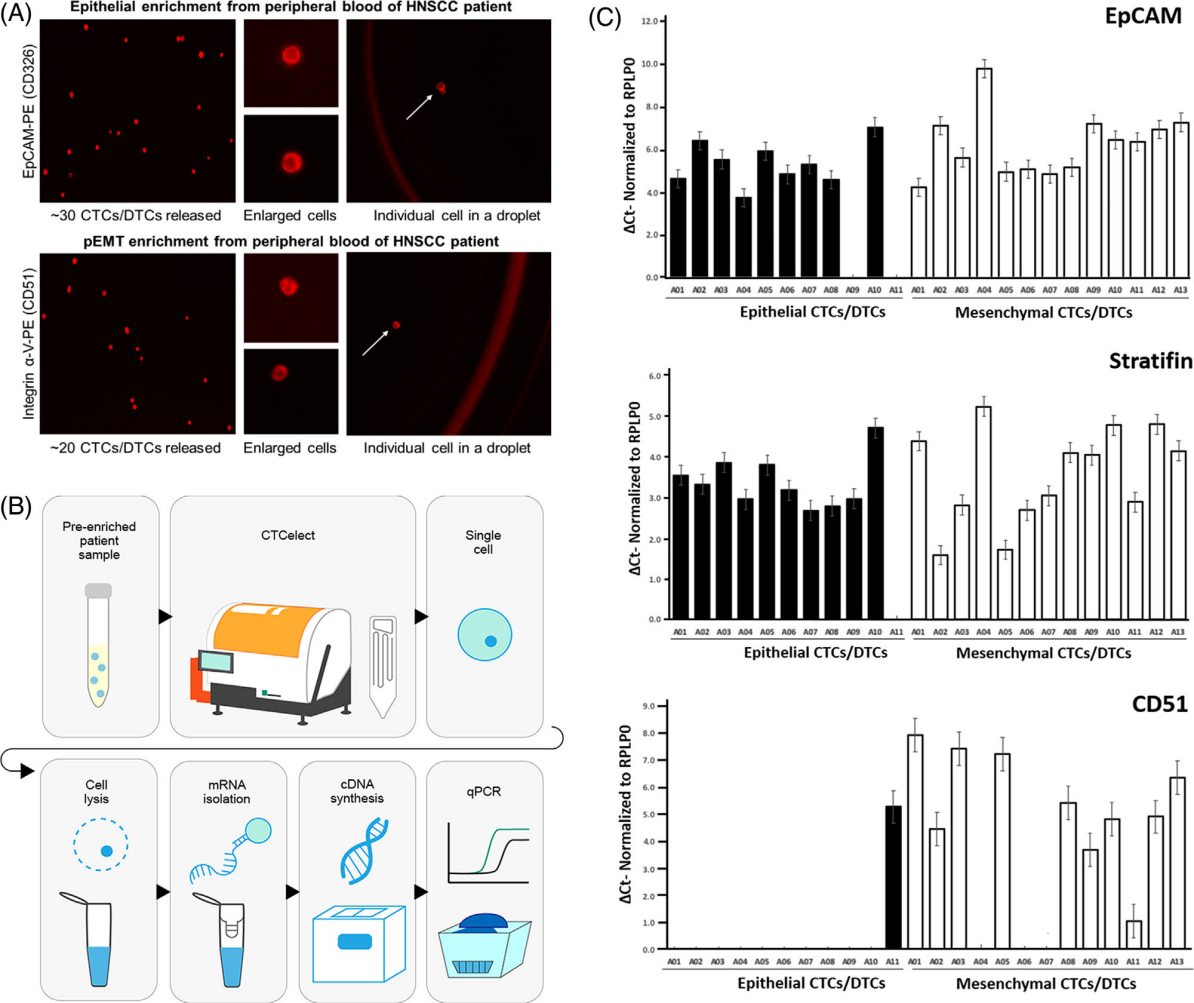
RT‐qPCR of CTC isolates after *CTCelect* single cell dispensing from pre‐enriched HNSCC patient blood. (A) Immunofluorescent staining of potential pre‐enriched CTCs/DTCs from HNSCC patient blood. Pre‐EMT CTCs were enriched with EpCAM beads and stained with EpCAM‐PE Ab from a 7.5 mL blood sample (left top). Integrin α‐V, CD61, CD106 beads and Integrin‐PE staining was used for partial/post‐EMT CTCs (left bottom). Cells were released from the beads by DNase I cleavage. The pre‐enriched samples were pipetted in the microfluidic cartridge of *CTCelect* and beads‐free CTCs were then single cell isolated in droplets by means of the *CTCelect* μCS subunit. The set of images on the right side shows potential single CTCs in droplets highlighted with white arrows and the droplet outlines. (B) Workflow of single cell RT‐qPCR. (C) Relative mRNA expression encoding EpCAM, Stratifin and CD51 normalized to β‐actin (RPLP0) by RT‐qPCR in epithelial and mesenchymal CTCs. Single cell total RNA was extracted from dispensed droplets and reverse transcribed into cDNA. cDNA was aliquoted and qPCR was conducted with one cDNA aliquot per well amplifying target nucleic acids respectively for EpCAM, CD51, Stratifin and β‐actin as positive control of epithelial‐like CTCs (pre‐EMT enrichment) and mesenchymal‐like CTCs (pEMT enrichment). Relative expression was calculated from triplicates using the ΔC_T_ method and is displayed as means with SD

Tumor‐specific single cell dispensing was investigated in peripheral blood of HNSCC patients. According to method 2.5 b), manual immunomagnetic enrichment was performed with EpCAM beads for pre‐EMT CTCs and Integrin α‐V, CD61, CD106 beads for partial/post‐EMT CTCs. Pre‐enriched samples were then detached from the beads and labeled with either EpCAM‐PE or Integrin α‐V‐PE staining (Figure 5A). In this patient, 30 EpCAM^+^ CTCs and 20 CD51^+^ CTCs were detected in 7.5 mL blood with fluorescence microscopy, respectively. Pre‐enriched and labeled samples were pipetted in the *CTCelect* chip and then automatically processed for single cell isolation in droplets (Figure 5B).

The successful single CTC isolation from patient blood by means of the microfluidic *CTCelect* unit was confirmed using RT‐qPCR. 11 epithelial‐like and 13 mesenchymal/pEMT‐like CTCs were isolated and singly dispensed from the pre‐enriched 7.5 mL blood samples of the same HNSCC patient (Figure 5B). RT‐qPCR was performed to detect target RNAs encoding EpCAM, Integrin α‐V, Stratifin and β‐actin as positive control. C_T_ values for each well were determined and relative mRNA expression was normalized to β‐actin (Figure 5C).

Samples with a C_T_ < 40 for β‐actin were identified as “positive” (droplet contained a cell) and samples with a C_T_ < 40 for β‐actin *and* at least one of the other three markers as “CTC‐positive” (droplet contained a potential CTC). False‐positive results were identified via melt curve analysis and excluded from the calculations. In this regard, 100 % of the dispensed droplets were CTC‐positive, since all of the 11 epithelial‐like and 13 mesenchymal‐like *CTCelect* samples had a C_T_ < 40 for β‐actin, EpCAM and Stratifin RNA at comparable levels. Additionally, all of the mesenchymal‐like CTCs were positive for CD51 RNA with sample A01 showing the highest an 8‐fold level of CD51 compared to β‐actin. In contrast, only 4 epithelial‐like CTCs (A03, 04, 10, 11) contained detectable CD51‐related nucleic acid and A11 even exhibited higher CD51 than EpCAM RNA. The 13 mesenchymal/pEMT‐like CTCs also significantly contained EpCAM RNA of at least 4‐fold greater than the housekeeping RNA.

**FIGURE 6:**
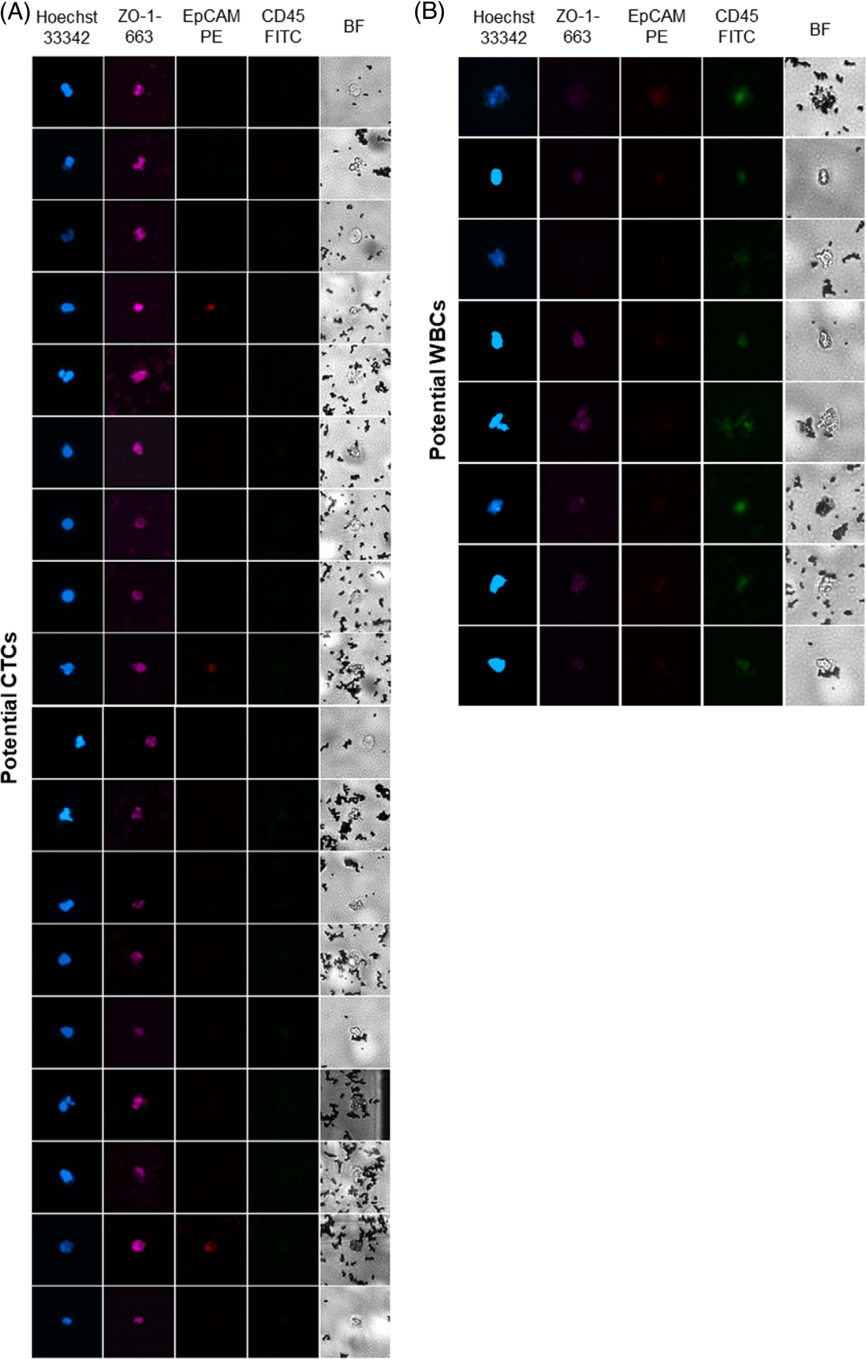
Immunostaining of potential CTCs and WBCs after CTCelect isolation. Dispensed cells were pooled together and subsequently stained with cell core dye Hoechst33342 (blue), ZO‐1 as epithelial marker (magenta) and CD45 as WBC marker (green). Residual EpCAM staining from CTCelect isolation is displayed in red. Cells were identified as (A) potential CTCs if Hoechst^+^/ZO‐1^+^/CD45^−^ and (B) as WBCs if Hoechst^+^/CD45^+^

Following method 2.5 c), completely automated CTC isolation from 7.5 mL HNSCC patient blood was performed. The dispensed droplets were pooled and stained using Hoechst33342 cell core staining, ZO‐1 as epithelial marker for potential CTCs and CD45 as negative control for contaminating WBCs. 18 potential isolated CTCs and 9 contaminating WBCs could be detected using fluorescence microscopy (Figure 6).

## 4 DISCUSSION

From the patient's point of view, single CTC isolation from peripheral blood is the least invasive procedure to evaluate tumor heterogeneity based on epigenomic, genetic or transcriptional markers and is therefore more time effective and reproducible than assays drawn from primary tumor tissue. This study covered a profound biological validation of the design, biotechnological effort and engineering concept of the *CTCelect* platform for automated single cell isolation from whole blood. *CTCelect* helps to identify, enrich, isolate and analyze CTCs and their subpopulations. Many researchers suggested that the precise characterization of CTCs will facilitate estimating both metastization patterns and outcome, driving clinical decision‐making and surveillance strategies [16].

For these purposes, the subunits of the system were separately tested and evaluated. Concerning cell enrichment, *CTCelect* provided recovery rates from medium of 68 ‐ 100 % in breast cancer single cells and 28% to 88 % in cultured squamous cell carcinoma cells. Further, 65% to 85 % of MCF‐7 cells and 20% to 55 % of SCL‐1 cells could be enriched from 7.5 mL whole blood from healthy donors (Figure 3). In comparison, Chudziak and colleagues determined 69.5 % enrichment efficiency of spiked lung cancer cells in their marker‐free CTC enrichment device Parsortix although it has to be mentioned that the sample input only ranged from 0.5 to 4 mL volume [17]. We extensively characterized the enrichment sub‐unit in our previous study before it was implemented in the overall CTCelect platform [18]. Similar to manual enrichment, there was no significant loss of cells due to vibrational disruption or tube surface adhesion. The enriched cells were accurately detected using fluorescent microscopy. More detailed, decreased enrichment efficiency by 15 % (MCF‐7) and 13 % (SCL‐1) in donor blood compared to culture medium was noted. As it has previously been postulated that the magnetic susceptibility in blood is significantly less negative than in aqueous solutions like medium [19, 20], we consequently hypothesize a generally lower magnetic force attracting immunomagnetic beads in whole blood. In addition, a steric competition with the abundant blood cell background could possibly prevent a proportion of the target CTCs from binding beads and being magnetized. Another important aspect influencing cell recovery rates is that cell deformability, viscoelasticity, and stability (density of actin filaments etc.) could play a role for robustness against shear stress in automated pipetting and these characteristics will be different among various cell types. Comparing the two cell lines, MCF‐7 cells were detected at higher rates than SCL‐1 cells. The density of antigens blocking phagocytosis could be higher on the cell surface of MCF‐7 cells and therefore be more favorable for spike‐in experiments. Breast cancer‐associated HER2 and lymphoma‐related CD47 exhibit a “*please don't eat me*” signal to macrophages [21, 22]. Besides that, Figure 3B showed that when the number of spiked SCL‐1 cells is higher than 25, there is an inconsistency between observed and expected cell count. This however does not suggest that the platform is not efficient in recovering larger numbers of CTCs as according to the results, the enrichment efficiency of MCF‐7 cells followed a linear trend at higher expected cell counts. In our recently published work, we screened several melanoma and carcinoma cell lines (MV3, SCL‐1, SCL‐2, BLM, patient‐derived HNC cells) and targets (MCSP, EpCAM, cell surface vimentin) to find a suitable candidate for the testing of microfluidic platforms. The enrichment fluctuation appeared frequently depending on the cell line and the sample volume also in manual enrichment. Further, we observed a similarly efficient cell line‐dependent recovery rate at even higher cell counts of 1000 cells for that is, A431 cells using the same beads [18]. The practicability of spike‐in experiments to test novel CTC platforms, in cell line models without evolutionary pressure in vitro combined with intracellular synthetic dyes, remains discussable and limits the study design. Nevertheless, these prevalidations are inevitable to investigate the proof‐of‐concept and invented design of the device. In this context, it has to be centered that the actual CTC count in a cancer patient has a multifactorial nature and therefore represents a dynamic measure with discrepancies.

For a successful singularization of the CTCs in the microfluidic cartridge and a high purity of the single cell dispensing it is also important to reduce the amount of WBC contamination during immunomagnetic enrichment. Thus, the automated enrichment process was characterized in terms of leukocyte by‐catch. Enriched samples of 7.5 mL whole blood from healthy donors were labeled with WBC marker CD45‐FITC and roughly 3.700 CD45 positive PBMC were detected via flow cytometry (Figure 3E). Healthy adults normally have a wide range of 3 ‐ 10 million leukocytes per mL blood [23], which concludes a by‐catch reduction of 1:10^5^ by means of *CTCelect* enrichment. Other devices like marker‐dependent CellSearch® or Isoflux™ and label‐free ScreenCell™ or ClearCell showed similar contaminations of 10^2^–10^4^ blood cells while authors also discussed a patient‐dependent discontinuity in leukocyte by‐catch using Parsortix [17, 24]. Their approaches often provide a final sample pooled with WBCs while subsequent marker‐based single cell detection in *CTCelect* further purifies the CTC fraction for compatible molecular analysis.

Combined enrichment with single cell detection and dispensing in one assay resulted in only minor cell losses with recovery rates of almost 60 % for both MCF‐7 and SCL‐1 cells from culture medium in the model system. Even from 7.5 mL whole blood, single cells were isolated at a probability of 57 % (MCF‐7) and 40 % (SCL‐1) (Figure 4A). The determined recovery rates from untreated whole blood at a high purity grade of less than 10 % probability to dispense a WBC with a CTC [25] showcases a fine parameter balance of *CTCelect* compared to other CTC isolation microdevices (Table 2). For example, studies on devices using hydrodynamics, size‐based filtration or dielectrophoresis showed indeed cell recovery rates of 70% to 85 % but were only able to process approximately 1 mL/h or lacked CTC isolate purity [5]. In fact, at a flowrate of 2.25*10^−15^ L/s and 5 ms nozzle‐emptying time, it is theoretically possible to detect, but not dispense, 200 events per second and 4*10^13^ cells/chip in 500 µL. The current dispensing limitation is the 500 ms drive time of the object table and the 96‐well plate format. *CTCelect* manages a moderate sample throughput of 3.3 mL/h with the current run time of around 2.3 h and 7.5 mL input volume. Additionally, most of the available systems require either sample transfer between different devices or blood pre‐processing like density gradient centrifugation for buffy coats. Pre‐enriched samples or yielded mononuclear phases of blood to specify the respective research needs can also be sampled in the herein discussed platform. To our knowledge, the possibility to use whole blood samples for automated single cell dispensing of rare cells in only one marker‐based device to make them available for corresponding analyzes stands alone.

**TABLE 2:** Comparison between CTCelect and different CTC isolation technologies.^1^

	**CTCelect**	**DEPArray**	**CellSearch**	**Sievewell**	**Parsortix**
Sample type	Whole blood, cell suspension	Cell suspension	Whole blood, leucapheresis	Cell suspension	Whole blood, cell suspension
Automated isolation of viable cells	Yes	yes	no	manually	Yes
Automated optical counting	Yes	setup	Yes	Yes	No
Isolation method	Marker	Electrophoresis, marker	Marker	Marker, size	Size, compressibility
Single cell dispensing	yes	yes	No	CellCelector	No
Isolation efficiency	Enrichment 38.8% to 72 % Dispensing 72.8 % Isolation 40% to 56.7 %	99.7 %^a)^	93 %^c)^ 81 %^d)^	n. a.	98 %^g)^ > 80 % (7.5 mL blood)^h)^ 30% to 70 % (1 mL blood)^h)^ 37 %^i)^
Purity (WBC contamination)	66 ‐ 90 %	100 %^a)^	800 WBCs/sample^e)^	n. a.	97 %^g)^ 29 %^h)^
Throughput	4*10^11^ cells/chip^2^; 96 wells (3.3 mL/h)	10‐10,000 cells/chip^b)^	n. a.	370,000 cells/chip^f)^	n. a.
Working volume	0.3–10 mL	n. a.	7.5 mL	0.5–2 mL/chip	10 mL
Cycle duration	2.25 h	2 ‐ 3 h	n. a.	n. a.	n. a.

^1^ *No claim to be exhaustive*.

^a)^ Di Trapani et al. 2018. Cytometry. Part A, 93(12), 1260–1266.

^b)^ http://www.siliconbiosystems.com/deparray‐technology‐faqs.

^c)^ https://documents.cellsearchctc.com/pdf/e631600006/e631600006_EN.pdf.

^d)^ Riethdorf et al. 2007. Clin Cancer Res 13(3), 920–928.

^e)^ Sieuwerts et al. 2009. Breast Cancer Res Treat 118, 455.

^f)^ https://www.sievewell.com/product‐information.

^g)^ Ciccioli et al. 2021. ANGLE plc AACR 2021 Virtual Meeting. https://angleplc.com/wp‐content/uploads/EMT‐assay‐poster‐AACR‐2021‐final.pdf [2021‐09‐10].

^h)^ Chudziak et al. 2014. CRUK – Manchester, NCRI conference 2014. https://angleplc.com/wp‐content/uploads/CRUK‐Manchester‐poster‐1.pdf [2021‐09‐10].

^i)^ Gorges et al. 2014. The University Medical Center Hamburg‐Eppendorf (UKE), ACTC Conference. https://angleplc.com/wp‐content/uploads/Angle_Hamburg_MC_poster_ebook‐2.pdf [2021‐09‐10].

^2^ *Not dispensable. Current limitation: 96‐well plate; drive duration of object table: 500 ms*.

The workflow for downstream single cell RT‐qPCR was simplified to immediate single cell lysis on the well plate. Direct reverse transcription in the cavities of the well plate coming from the device as well as the assay implementation to release detachable beads in the enrichment unit are subjects of future work. This evaluation however included the earliest results of the *CTCelect* device towards isolating single CTCs from a patient with metastatic head and neck squamous cell carcinoma in a clinical environment. In these patient samples, 30 EpCAM^+^ CTCs and 20 CD51^+^ CTCs were detected via fluorescence microscopy (Figure 5A). Microfluidic cell singularization enabled single cell RT‐qPCR and allowed a distinction in 11 pre‐ and 13 pEMT CTC subtypes on the basis of different tumor‐related markers (Figure 5C). Hence our findings evidence the potential of *CTCelect* to depict cancer plasticity. IVD companies, that is, QIAGEN N.V., offer non‐automated CTC isolation/PCR platforms to characterize common tumor entities such as lung, prostate and breast cancer, but not for HNSCC diagnostics. Further, CTC liquid biopsy in HNSCC could elucidate crucial information on early assessment of treatment measures and effectiveness with respect to the detection of micrometastases at the initial diagnosis, complementing well‐established ctDNA characterizations. Mouliere et al. recently detected chromosomal mutations of solid tumors in the blood by whole genome copy number variation analysis [26]. Encompassing studies on ctDNA and CTC characterizations have been carried out [27] but, especially in head and neck cancers, the respective literature landscape is sparsely settled and to be addressed in the future with the suggested data.

It is obvious that immuno‐biochemical techniques targeting antigens like EpCAM are not infallible for all tumor entities or heterogeneous CTC populations, especially CTCs undergoing epithelial‐mesenchymal transition (EMT) which is closely linked to invasive metastasis. Against that, mixtures of antibodies like integrin subtypes, cell surface vimentin or stem cell markers are of assistance to improve the enrichment of partial/post‐EMT mesenchymal‐like CTCs. Besides these affinity‐based techniques to enrich and isolate CTCs, the physical methods base on distinction in cell size, density or even plasticity. Due to the fact that CTCs are a heterogeneous population in blood, they have some characteristics in common with healthy mononuclear blood cells. Thus, methods based on filtration, centrifugation or size exclusion may not be used for a standard clinical test system. Further, it is important to mention that there are other obstacles, i.e. the purity of the CTC fraction, before realizing subsequent applications such as single cell sequencing or copy number variation studies.

The certainly limited patient screening in this study clearly demonstrated the feasibility of downstream applications. The platform enabled fully‐automated CTC isolation from HNSCC patient blood and immunofluorescent identification of 18 potential CTCs for proof‐of‐concept (Figure 6). Several researchers have stated that the presence of CTCs in the blood is of prognostic relevance for overall and progression‐free survival in patients with head and neck cancer [28] and that HNSCC exhibits early stage micrometastatic sites and severe intra‐tumoral heterogeneity which is closely linked to poor disease outcome. In this context, an important advantage of CTC/DTC isolation over imaging technologies is that minimal residual disease (MRD), for example DTC micrometastases, are undetectable by the latter. Especially bone marrow is intensively studied as a reservoir for dormant DTCs with the capacity to re‐enter the circulatory system and trigger MRD in distant tissues [29]. It is easily plausible to broaden the applicability of the *CTCelect* device to process liquid biopsies from bone marrow or dissolved lymph node resections. All of these aspect stress the necessity of an improved cell biological understanding to foremost provide a good care for HNSCC patients. High‐resolution of cell heterogeneity, metastatic invasiveness and evolutionary pressure on cancer cells is key to precision medicine. To conclude, the presented results demonstrate the robust technical performance of *CTCelect* and its feasibility as a novel tool for liquid biopsy to make CTCs available for corresponding examinations on a single cell level with respect to head and neck squamous cell carcinoma.

## CONFLICTS OF INTEREST

The authors have declared no conflicts of interest. All authors have read and agreed to the published version of the manuscript.

## Supporting information

Supporting Information

## Data Availability

The data that support the findings of this study are available on request from the corresponding author. The data are not publicly available due to privacy or ethical restrictions.
