# Supplementary material for: Characterization of a novel microfluidic platform for the isolation of rare single cells to enable CTC analysis from head and neck squamous cell carcinoma patients
Source: Eng Life Sci. 2022 Apr 6;22(5):391–406. doi: 10.1002/elsc.202100133 (PMC9077830; doi:10.1002/elsc.202100133)
Supplement: Supplementary file 1 — Supporting Information [file ELSC-22-391-s001.pdf]

Supporting information

Characterization of a novel microfluidic platform for the isolation of rare single cells to enable CTC analysis from head and neck squamous cell carcinoma patients

Janis Stiefel, Ashwin Sriram, Christian Freese, Sabine Alebrand, Nalini Srinivas, Christoph Sproll, Madita Wandrey, Désirée Gül, Jan Hagemann, Jürgen C. Becker, Michael Baßler

Wiley *Engineering in Life Sciences*, 2021.

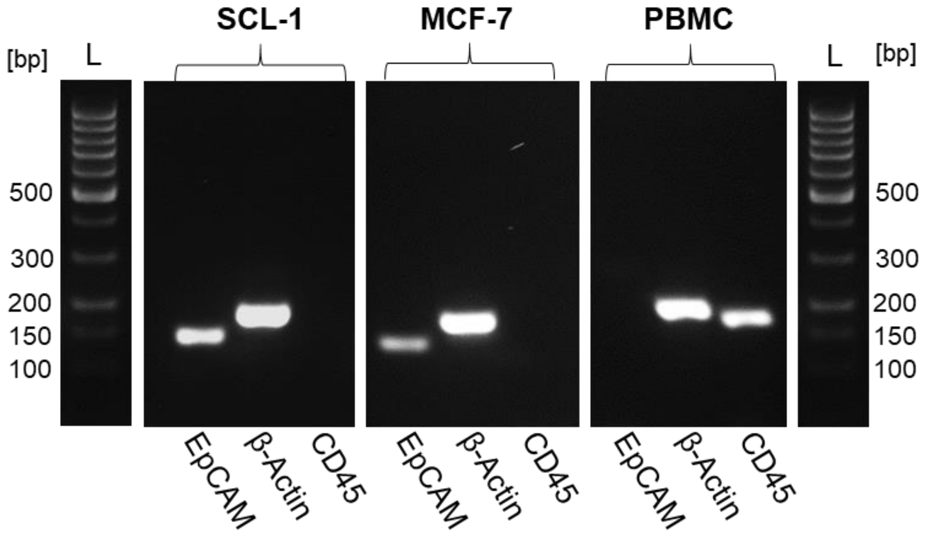

Figure S1: Gel electrophoresis of RT-qPCR products from single cell RNA to confirm EpCAM transcripts in the SCL-1 and MCF-7 cell line. Total single cell RNA was isolated using RNeasy Micro Kit (QIAGEN) with an adapted protocol lysing one cell. cDNA was synthetized with Sensifast cDNA synthesis Kit (Bioline). qPCR was performed with Quantifast qPCR Kit (QIAGEN). EpCAM product was expected at a size of 136 bp. β-Actin (176 bp) served as reference transcript. RT-qPCR towards leukocyte marker CD45 (159 bp) of one peripheral blood mononuclear cell RNA from a healthy donor was used as negative control. Primers were designed with Primer-BLAST (National Institutes of Health; https://www.ncbi.nlm.nih.gov/tools/primer-blast/).

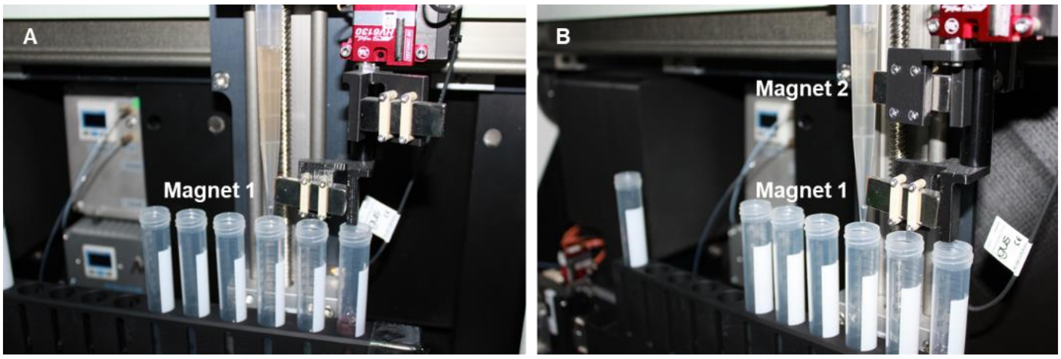

Figure S2: Automated enrichment in the CTCelect device. A) After each washing step, bead-cell pellets were captured in the 10 mL pipet tip by placing magnet 1 on one side of the pipet tip. B) When washing in larger volumes (5 mL), immunomagnetic enrichment was additionally performed using the upper magnet 2.

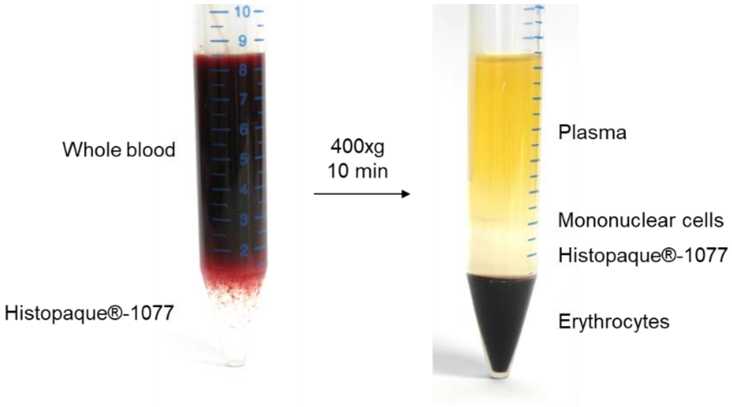

Figure S3: Buffy coat of peripheral blood mononuclear cells from a healthy donor. 1 mL Histopaque®-1077 (Merck, Darmstadt, DE) was coated with 7.5 mL whole blood and centrifuged for 10 min at 400xg. The plasma was discarded. The PBMC phase was collected, resuspended 1:1 in PBS and centrifuged for 5 min at 400xg. The cell pellet was resuspended in 1 mL PBS for further experiments.
